# The crowding-out effect of tobacco expenditure on household spending patterns in Bangladesh

**DOI:** 10.1371/journal.pone.0205120

**Published:** 2018-10-09

**Authors:** Muhammad Jami Husain, Biplab Kumar Datta, Mandeep K. Virk-Baker, Mark Parascandola, Bazlul Haque Khondker

**Affiliations:** 1 Global Noncommunicable Diseases Branch, Division of Global Health Protection, Center for Global Health, Centers for Disease Control and Prevention, Atlanta, GA, United States of America; 2 National Cancer Institute, National Institutes of Health, Rockville, MD, United States of America; 3 Department of Economics, University of Dhaka, Dhaka, Bangladesh; RTI International, UNITED STATES

## Abstract

**Background:**

Tobacco consumption constitutes a sizable portion of household consumption expenditure, which can lead to reduced expenditures on other basic commodities. This is known as the crowding-out effect. This study analyzes the crowding-out effect of tobacco consumption in Bangladesh, and the research findings have relevance for strengthening the tobacco control for improving health and well-being.

**Methods:**

We analyzed data from the Bangladesh Household Income and Expenditure Survey 2010 to examine the differences in consumption expenditure pattern between tobacco user and non-user households. We further categorize tobacco user households in three mutually exclusive groups of smoking-only, smokeless-only, and dual (both smoking and smokeless); and investigated the crowding-out effects for these subgroups. We compared the mean expenditure shares of different types of households, and then estimated the conditional Engel curves for various expenditure categories using Seemingly Unrelated Regression (SUR) method. Crowding-out was considered to have occurred if estimated coefficient of the tobacco use indicator was negative and statistically significant.

**Results:**

We find that tobacco user households on average allocated less in clothing, housing, education, energy, and transportation and communication compared to tobacco non-user households. The SUR estimates also confirmed crowding-out in these consumption categories. Mean expenditure share of food and medical expenditure of tobacco user households, however, are greater than those of tobacco non-user households. Albeit similar patterns observed for different tobacco user households, there were differences in magnitudes depending on the type of tobacco-use, rural-urban locations and economic status.

**Conclusion:**

Policy measures that reduce tobacco use could reduce displacement of commodities by households with tobacco users, including those commodities that can contribute to human capital investments.

## Introduction

Tobacco use is among the leading causes of preventable premature deaths and disabilities, globally. Smoking tobacco harms nearly every organ of the body, impairs immune function, causes inflammation, and increases risk for deaths from all causes in men and women [1]. Smokeless tobacco use can cause cancer of mouth, esophagus, and pancreas, increase risks for early delivery and stillbirth during pregnancy, lead to nicotine addiction and nicotine poisoning in children, and increase risk of deaths from cardiovascular diseases [2, 3]. The tobacco epidemic kills more than 7 million people each year globally; and more than two-thirds of the more than 1 billion smokers worldwide live in low and middle-income countries [4].

In addition to the deleterious health effects, the economic impacts of tobacco consumption at the individual, family, and national levels are also a major concern. Tobacco use causes enormous economic costs for individuals and families due to illness, disability, premature deaths, forgone consumption and investments, and bad consumption choices [2, 3, 5]. At the macro-level, tobacco use contributes to less healthy workforce, lost productivity, high healthcare costs, strains in healthcare systems, degradation of natural environment, and health disparities [5, 6, 7].

Published studies on the nexuses among tobacco use, nutrition, human capital investments, and poverty demonstrate that expenditures on tobacco smoking constitute a significant portion of household budget, which can lead to reduced spending on other basic commodities like food, health, education, housing, transport, energy etc. [8, 9, 10, 11, 12, 13, 14]. This phenomenon is known as the crowding-out effect, which in turn, may exacerbate the effects of poverty, including the impact on nutritional status of children [15, 16, 17, 18].

Tobacco use is pervasive in Bangladesh, where 58% of men and 29% of women use tobacco in smoking or smokeless form [19]. On average, a tobacco-user household spent nearly 392 Bangladesh Taka (BDT) per month in 2010, constituting 4.1% of the household budget. This expenditure share is much higher than many other food consumption items, including fruits, legumes and beans, oil and fat, milk, sugar, and eggs [20]. Husain et al. [20] suggested that, in Bangladesh, the tobacco expenditure-shift could translate to an additional 4.6 to 7.7 million food-energy malnourished persons meeting their caloric requirements. Previously, Efroymson et al. [8] asserted that, in Bangladesh, the average male cigarette smoker spends more than twice as much on cigarettes as compared to combined expenditures on clothing, housing, health and education; that a typical poor smoker could add over 500 calories to the diet of one or two children by eliminating daily tobacco expenditure; and that an estimated 10.5 million malnourished people could have an adequate diet if money on tobacco were spent on food instead.

Previous literature on the consumption displacement attributable to tobacco use primarily considered smoking tobacco (e.g. cigarettes, bidi, and other forms of smoking tobacco). This paper presents an analysis of household level tobacco use by different tobacco use types. Husain et al. [20] analyzed smokeless tobacco use that showed the potential trade-offs between nutrition intake and tobacco use. To our knowledge, this is the first study that contributes to the tobacco use crowding-out literature by examining how the household expenditure patterns for smoking-, smokeless-, and dual- (both smoking and smokeless) user households differ from tobacco non-user households, from the recent data in Bangladesh. The findings of the study highlights the potential trade-offs between tobacco expenditure and all other different household expenditure categories, and provides further evidence for tobacco control as an essential component for improving health and well-being.

## Methods

### Data

We used the Bangladesh Household Income and Expenditure Survey (HIES, 2010). The HIES is a nationally representative survey, consisting of 12,240 households (7,840 rural and 4,400 urban), and the major source of socio-economic information at the household level in Bangladesh. The HIES methodology and survey design details are available in published reports [21, 22]. HIES collects consumption information on over 300 food and non-food items, which were separated into 11 mutually exclusive subgroups (tobacco, food, clothing, housing, education, health, lifestyle and hygiene, energy and utility, transport and communication, entertainment, and miscellaneous).

The daily food consumption quantities and expenditure values were collected for 14 days over seven visits with two days’ recall. There are some weekly food-items (e.g. various spices), information on which were collected for two weeks. Daily consumption of 122 food items under 14 broad categories, and weekly consumption of 11 food items under the spices category were reported in the survey. Expenditures on tobacco and tobacco products, including cigarettes, tobacco leaf, bidies, and gul were recorded in the daily-food-consumption section, whereas some other smokeless products (i.e. betel leafs and chew goods) were recorded in the weekly-food-consumption section. These tobacco products were grouped into smoking and smokeless categories (i.e. smoking only, smokeless only, and dual). The consumption module recorded expenditures on non-food items either as monthly expenditure (e.g. energy, life style and hygiene, transport and communication), or yearly expenditure (e.g. clothing, housing, education, health) [21, 22]. For our analysis, all consumption expenditures are converted in average monthly expenditures.

### Household tobacco use

A household was considered tobacco-user if that household reported any expenditure on tobacco. The expenditure can be incurred by a single person, or by multiple persons, and can include smoking-, smokeless-, or dual- tobacco user(s). Comparisons were made between tobacco non-user households and ‘any’ tobacco user households; and also with mutually exclusive smoking-only, smokeless-only, and dual (both smoking and smokeless) tobacco user households. HIES data allowed this unique categorization of household groups by tobacco use types. This kind of classification distinguishes our study from previous literature by highlighting differential tobacco use determinants associated with household characteristics, and in terms of econometric model specifications.

According to the Bangladesh Global Adult Tobacco Survey (GATS 2009), at the individual level, tobacco use is more prevalent in rural areas (45.1%) than urban areas (38.1%), and in the lowest quintiles of socioeconomic status (SES) (55.6%). At the individual level, 23.0% (21.9 million persons) of adults aged 15 years or above smoke tobacco, and 27.2% (25.9 million) use smokeless tobacco. For males, 44.7% and 26.4% use smoking and smokeless tobacco, respectively. The corresponding prevalence for females are 1.5% and 27.9% for smoking and smokeless tobacco, respectively [19].

In Table 1, the distribution of households by tobacco use type for the top and bottom consumption expenditure quintiles shows contrasting prevalence of smoking and smokeless household tobacco use. The pattern also differs by rural and urban populations. At the national level, 69.8% of the households reported spending money on tobacco, whereas the household level prevalence in urban areas is much lower (56.7%) than in rural areas (74.6%). Household level prevalence of smoking-only tobacco is higher in urban area than in rural area (19.2% vs. 16.5%); in contrast, prevalence of smokeless-only tobacco is higher in rural areas than in urban areas (27.9% vs. 20.9%). This contrasting pattern is more striking for the households in the top expenditure quintile. 18.6% of households in top quintile in urban areas reported spending on smoking-only tobacco compared to 11.6% of top quintile in rural areas. For the smokeless-only tobacco use in the top quintile households the percentages are 21.7% and 31.4% for urban and rural areas, respectively.

**Table 1:** Distribution of households by tobacco use type and household expenditure per capita quintiles.

	All (%)	Bottom Quintile (%)	Top Quintile (%)
**National**			
Households do not consume tobacco	30.2	28.0	37.0
Households consume any tobacco	69.8	72.0	63.0
*Smoking only tobacco use*	*17*.*3*	*19*.*7*	*15*.*1*
*Smokeless only tobacco use*	*26*.*0*	*24*.*7*	*28*.*2*
*Dual (both) tobacco use*	*26*.*5*	*27*.*6*	*19*.*8*
Number of Households	33,028,016	6,605,682	6,604,107
Number of Households (Sample)	12,240	2,444	2,495
**Urban**			
Households do not consume tobacco	43.3	34.3	50.6
Households consume any tobacco	56.7	65.7	49.4
*Smoking only tobacco use*	*19*.*2*	*22*.*9*	*18*.*6*
*Smokeless only tobacco use*	*20*.*9*	*20*.*2*	*21*.*7*
*Dual (both) tobacco use*	*16*.*6*	*22*.*5*	*9*.*0*
Number of Households	8,860,148	1,773,390	1,769,080
Number of Households (Sample)	4,400	1,219	709
**Rural**			
Households do not consume Tobacco	25.4	28.5	24.4
Households consume any tobacco	74.6	71.5	75.6
*Smoking only tobacco use*	*16*.*5*	*19*.*1*	*11*.*6*
*Smokeless only tobacco use*	*27*.*9*	*25*.*5*	*32*.*6*
*Dual (both) tobacco use*	*30*.*1*	*26*.*9*	*31*.*4*
Number of Households	24,167,867	4,835,249	4,831,015
Number of Households (Sample)	7,840	1,563	1,580

**Note:** The distributions were derived applying relevant weights to take into account the complex survey design. Shares of smoking only-, smokeless only-, and dual (both)- tobacco use add up to “Households consuming any tobacco”.

Comparison of the distribution of household tobacco use between bottom and top quintiles revealed that, at the national level, 72.0% of households in the bottom quintile reported spending on any tobacco compared to 63.0% in the top quintile. This pattern is similar for smoking-only tobacco use at the national, urban, and rural populations; i.e. percentage of households in the bottom quintile is higher than the top quintile counterparts. However, this pattern is reversed for smokeless-only tobacco use at the national level (24.7% for bottom quintile vs. 28.2% for top quintile); and for urban (20.2% vs. 21.7%), and rural (26.9% vs. 31.4%) areas.

Table 2 presents descriptive statistics on some socio-economic and demographic variables. All the descriptive statistics were generated taking into account the complex survey design and using appropriate survey weights. It is evident that the proportion of male among adults in smoking-only tobacco user households are higher compared to smokeless-only tobacco user households (50.9% vs. 43.2%, respectively). Also, only 3.0% of the smoking-only households are headed by a female member, compared to 20.0% of smokeless-only households headed by a female member. The proportion of household heads with no education is higher among tobacco user households compared to non-user households (50–63% vs. 45%). Table 2 also suggests larger average household size and lower average per capita income for tobacco user households. The proportion of household heads with higher degrees is relatively less in tobacco user households; and larger percentage of smokeless and dual user households have elderly present in the household.

**Table 2:** Descriptive statistics on household socio-economic and demographic variables.

	Household tobacco use type			
	Tobacco non-user	Smoking-only tobacco user	Smokeless-only tobacco user	Dual (Smoking and smokeless) user
Household income per capita (BDT)	3311 (3044, 3578)	2503 (2317, 2688)	2599 (2452, 2746)	2256 (2130, 2382)
Food expenditure per capita (BDT)	1340 (1293, 1388)	1261 (1213, 1308)	1330 (1286, 1374)	1240 (1206, 1274)
Non-food expenditure per capita (BDT)	1435 (1293, 1578)	1153 (1005, 1300)	1224 (1148, 1300)	943 (894, 992)
Tobacco expenditure per adult (BDT)	0.0	109 (101, 117)	99 (91, 107)	193 (181, 205)
Household Size (number of people)	4.0 (3.9, 4.1)	4.4 (4.3, 4.5)	4.5 (4.4, 4.6)	5.1 (5.0, 5.2)
Proportion of male among adult (%)	42.9 (42.0, 43.8)	50.9 (50.3, 51.4)	43.2 (42.4, 44.0)	50.8 (50.3, 51.2)
Proportion of children (%)	32.9 (32.0, 33.9)	34.2 (33.2, 35.3)	27.9 (27.0, 28.8)	31.9 (31.0, 32.9)
Presence of elderly (proportion)	0.17 (0.16, 0.19)	0.13 (0.12, 0.15)	0.36 (0.34, 0.38)	0.28 (0.27, 0.30)
Female-headed household (proportion)	0.23 (0.21, 0.25)	0.03 (0.02, 0.03)	0.20 (0.19, 0.22)	0.04 (0.04, 0.05)
Household Head's Education (Proportion)				
*No education*	0.45 (0.42, 0.48)	0.55 (0.52, 0.58)	0.50 (0.48, 0.53)	0.63 (0.61, 0.65)
*Primary*	0.14 (0.12, 0.15)	0.17 (0.15, 0.19)	0.17 (0.16, 0.18)	0.16 (0.14, 0.17)
*Secondary*	0.32 (0.30, 0.34)	0.25 (0.23, 0.28)	0.27 (0.25, 0.29)	0.19 (0.18, 0.21)
*Baccalaureate*	0.05 (0.04, 0.06)	0.03 (0.02, 0.03)	0.04 (0.03, 0.05)	0.01 (0.01, 0.01)
*Graduate/ Professional*	0.04 (0.03, 0.06)	0.01 (0.00, 0.01)	0.02 (0.01, 0.02)	0.01 (0.00, 0.01)

Note: BDT: Bangladesh Taka. 95% confidence interval in the parenthesis.

To explore the determinants of tobacco use by types, we used a logistic regression model that estimated the probability of a household using any tobacco; and a multinomial logistic regression that estimated the probabilities of a household using smoking-only, smokeless-only, or dual (both types) tobacco, given a set of socio-demographic characteristics.

$$P(T_{i}|X)=\frac{exp(x_{i}^{\prime }\beta_{j})}{1+exp(x_{i}^{\prime }\beta_{q})}$$ (1)

Eq (1) shows the logistic regression specification, where, *T*_*i*_ takes the value 1 if household *i* has positive tobacco expenditure, and 0 otherwise; **X** is a set of household level characteristics and other relevant control variables, including proportion of male among adult household members, proportion of children, presence of elderly (aged 60+), sex of household head, household size, household income per capita, religion, principal source of household income, level of education of household head, and district fixed effects.

$$P(T_{i}=t|X)=\frac{exp(x_{i}^{\prime }\beta_{j})}{1+\sum_{q=1}^{3}exp(x_{i}^{\prime }\beta_{q})}$$ (2)

Eq (2) is the multinomial logistic regression specification, where, *t* represents different categories of tobacco consumption. The base category here is ‘no’ tobacco-user. The results of logistic estimation are usually interpreted using marginal effects, as shown in Eq (3):

$$\frac{\delta P(T_{i}=t|X)}{\delta x_{ik}}=p_{ij}\left(\beta_{jk}-\sum_{q=1}^{3}\beta_{qk}p_{iq}\right)$$ (3)

Eq (3) is the marginal effect of the *k*^*th*^ explanatory variable, *X*_*ik*_, on the *t*^*th*^ response probability of household *i*. Average marginal effects are then obtained by taking arithmetic mean of the marginal effects as following:

$$AME_{tk}=\frac{1}{N}\sum_{i=1}^{N}\frac{\delta P(T_{i}=t|X)}{\delta x_{ik}}$$ (4)

Results from logistic regression show that probability of consuming any tobacco is on average lower for female headed households, and households with larger proportion of children; and higher for households with elderly members (Table 3). However, multinomial logistic regression results indicate that several of these findings could be different for different tobacco use types. Contrary to the lower probability of consuming any tobacco, probability of consuming smokeless tobacco-only is higher for female headed households. Likewise, probability of consuming smoking tobacco only is lower for households with elderly members, though probability of consuming any tobacco is higher for these households. These results suggest considerable heterogeneity in tobacco use across households, and thus justify our approach of analyzing the crowding out effect for different tobacco use types.

**Table 3:** Probability of tobacco consumption: average marginal effects of household demographics.

	Logistic	Multinomial Logistic		
	Any	Smoking only	Smokeless only	Dual
Proportion of males among adults	-0.00006 (-0.00066, 0.00054)	0.00078*** (0.00020, 0.00136)	-0.00177*** (-0.00236, -0.00117)	0.00115*** (0.00059, 0.00170)
Proportion of children	-0.00223*** (-0.00270, -0.00176)	0.00080*** (0.00034, 0.00126)	-0.00190*** (-0.00237, -0.00144)	-0.00114*** (-0.00163, -0.00065)
Female headed household	-0.20202*** (-0.23710, -0.16693)	-0.13473*** (-0.15446, -0.11500)	0.09133*** (0.05658, 0.12608)	-0.17513*** (-0.19800, -0.15225)
Presence of elderly (age 60+)	0.05951*** (0.03817, 0.08086)	-0.06875*** (-0.08861, -0.04890)	0.10122*** (0.07949, 0.12294)	0.02156** (0.00236, 0.04076)
Education^†^				
*Primary*	-0.02540** (-0.04787, -0.00292)	-0.00059 (-0.02276, 0.02157)	0.03446*** (0.01195, 0.05696)	-0.05834*** (-0.08108, -0.03560)
*Secondary*	-0.10549*** (-0.12933, -0.08165)	-0.02007* (-0.04057, 0.00042)	0.03133*** (0.01032, 0.05233)	-0.11530*** (-0.13384, -0.09676)
*Bachelor’s*	-0.20788*** (-0.26908, -0.14669)	-0.05217** (-0.09249, -0.01184)	0.08144*** (0.03255, 0.13033)	-0.23572*** (-0.27010, -0.20134)
*Graduate or Professional*	-0.31654*** (-0.38525, -0.24784)	-0.12491*** (-0.16116, -0.08866)	0.03419 (-0.02761, 0.09600)	-0.22264*** (-0.27150, -0.17377)
HH per capita income groups				
*700 to 999*	-0.00590	-0.01012	0.02514	-0.01974
	(-0.04306, 0.03127)	(-0.04127, 0.02103)	(-0.00996, 0.06023)	(-0.05154, 0.01206)
*1000 to 1499*	-0.00703	-0.01536	-0.01639	0.02442
	(-0.03983, 0.02576)	(-0.04236, 0.01163)	(-0.04732, 0.01454)	(-0.00604, 0.05488)
*1500 to 2499*	0.00208	-0.00994	-0.00767	0.01993
	(-0.03060, 0.03476)	(-0.03737, 0.01750)	(-0.03603, 0.02069)	(-0.00799, 0.04785)
*2500 to 4999*	-0.00515	-0.01764	0.00688	0.00843
	(-0.04127, 0.03097)	(-0.04882, 0.01354)	(-0.02538, 0.03913)	(-0.02449, 0.04136)
*5000 & more*	-0.02302	-0.01397	-0.01569	0.01361
	(-0.06550, 0.01946)	(-0.05241, 0.02448)	(-0.05337, 0.02198)	(-0.03010, 0.05732)
Household Religion				
*Hinduism*	-0.02580	0.01496	-0.04982***	0.01031
	(-0.06029, 0.00868)	(-0.01101, 0.04092)	(-0.07928, -0.02036)	(-0.01580, 0.03643)
*Other*	0.11571***	0.07703	-0.06707	0.11115***
	(0.05612, 0.17530)	(-0.02517, 0.17922)	(-0.16050, 0.02635)	(0.03405, 0.18825)
Household Size				
*3 to 5*	0.11429***	0.02554	0.02502*	0.07579***
	(0.07668, 0.15189)	(-0.00541, 0.05649)	(-0.00399, 0.05402)	(0.04892, 0.10265)
*6 to 9*	0.22007***	0.00652	0.06289***	0.16461***
	(0.17966, 0.26048)	(-0.02867, 0.04171)	(0.02808, 0.09769)	(0.13239, 0.19682)
*10 and more*	0.27773***	-0.05132	0.05405	0.28242***
	(0.21793, 0.33753)	(-0.11348, 0.01083)	(-0.01442, 0.12252)	(0.21318, 0.35166)
HH major source of income				
Agri Production	0.02948*	0.01490	-0.03243**	0.05410***
	(-0.00059, 0.05955)	(-0.00996, 0.03975)	(-0.06266, -0.00221)	(0.02593, 0.08227)
Non-agri Production	0.04984***	0.05189***	-0.03242**	0.03781***
	(0.01502, 0.08465)	(0.02127, 0.08250)	(-0.06234, -0.00250)	(0.01071, 0.06490)
Wage & Salary	0.00390	0.05358***	-0.06185***	0.02090*
	(-0.02165, 0.02945)	(0.03077, 0.07639)	(-0.08623, -0.03748)	(-0.00258, 0.04438)

Note

*** p<0.01

** p<0.05

* p<0.1. 95% confidence interval in the parenthesis.

^†^Education refers to the highest education level of the household head. ‘No’ education is the reference category.

### The crowding-out effect

The crowding out effect of tobacco expenditure entails reduced consumption of goods and services because of tobacco consumption. Depending on the level of analysis (i.e. individual or household), data availability, and methodological approach, the crowding-out effects have been reported as the differences in the mean expenditure shares of different consumption categories between tobacco users and non-users, and/or the marginal effects of tobacco expenditure on other commodities or services. The conceptual framework of this paper followed the most recent generation of empirical studies on the crowding-out impact of tobacco expenditure [10, 13, 14, 15, 16, 17]. However, the empirical strategy is most aligned with John, Ross, and Blecher [16] that defined the crowding-out effect as reduced consumption of goods and services as a result of tobacco consumption.

#### Conceptual framework

Households can report zero tobacco expenditures either because none in the household use any form of tobacco, even if they have adequate income; or households cannot afford tobacco products, given their income. The former explanation implies that there is a difference between the spending patterns of smoking and non-smoking households [14]. The crowding-out attribution of tobacco expenditure in displacing expenditure on other commodities comes with the assumption that a household that spends on tobacco decides on the quantity of tobacco to be purchased before deciding on the quantities of the other goods and services. Given this, household’s demand for a particular commodity is conditional on the household’s tobacco use status and the remainder of household income after spending on tobacco [10, 15]. Following the recent literature, we estimated and compared a set of Engel curves for tobacco non-user households with conditional Engel curves for tobacco user households for a common set of commodities. If, on average, the quantity demanded of a commodity for the typical tobacco user household is less (more) than the quantity demanded of the same commodity for a typical tobacco non-user household, then the difference can be attributed, ceteris paribus, to tobacco use [16].

#### Empirical strategy

In the first step of our empirical strategy, we compared the mean expenditure shares for the food and various non-food expenditure categories between households with and without tobacco expenditure using the t-test on the equality of means. Statistically significant differences in the expenditure dedicated to other commodities in the budgets of tobacco user households (i.e. any tobacco, smoking-only, smokeless-only, and dual user households) and non-user households indicate unadjusted crowding-out effect. However, these unadjusted differences in expenditure shares do not take into account households’ socioeconomic and demographic characteristics that may have influence on spending pattern.

Therefore, we formally tested the crowding-out hypothesis using multivariate regression analysis, which predicted the budget share allocation to each expenditure category according to tobacco use status, controlling for household-specific and other characteristics. To determine differences in spending patterns between tobacco user and non-user households, the regression models estimated conditional Engel curves for 10 expenditure categories using the Quadratic Almost Ideal Demand System (QUAIDS) developed by Banks, Blundell, & Lewbel [23]. Depending on household expenditure level, a particular consumption item could be either necessary or luxury. Use of QUAIDS allows a particular expenditure category to be either a luxury or a necessity by including a quadratic expenditure term in the specification [10, 23]. If household expenditure in one category is correlated with expenditures on other categories, the error terms in the Engle curve estimations are likely to be correlated, potentially leading to increased variance in the estimated coefficients and inefficient coefficient estimates. We addressed this by using Seemingly Unrelated Regression (SUR) model, which estimated all regression equations simultaneously using a Feasible Generalized Least Squares (FGLS) method [16].

Several studies on the crowding-out effect of smoking emphasized the potential endogeneity of total expenditure and smoking expenditure, and therefore used the instrumental variable (IV) method to obtain consistent and unbiased estimators [10, 14, 15, 17]. Those studies used income or total value of household assets as instruments for total expenditure, and adult sex ratio or female ratio in the household as instrument for expenditure on tobacco. While in this paper we instrumented household total expenditure by total income, we believe that adult sex ratio (or female ratio) does not satisfy the exclusion restriction requirements due to their potential correlations with other consumption items. More importantly, while adult sex ratio may explain smoking decision in a household, we found that the same is not true for the decision to spend on smokeless tobacco; instead we use proportion of males among adults and other household demographics as control variables in the regression specifications.

$$w_{ij}=\beta_{0}+\beta_{1}T_{i}+\beta_{2}\ln M_{i}+\beta_{3}{\left(\ln M_{i}\right)}^{2}+X\beta_{4}+\sum_{d=1}^{63}\gamma_{d}District_{id}+\varepsilon_{ij}$$ (5)

Eq (5) shows the regression specification for ‘any’ tobacco consumption, where, *w*_*ij*_ is household *i*'s expenditure share of category *j*. Expenditure shares are calculated after deducting expenditure on tobacco. *T*_*i*_ is a binary variable that takes the value 1 if household *i* consumes any type of tobacco, and 0 otherwise. *M*_*i*_ is monthly consumption expenditure of household *i*, excluding tobacco expenditure. **X** is a vector of household level characteristics; including presence of children aged under five, presence of children aged 5 to 14, presence of elderly (aged 60+), proportion of male among adult household members, household size, sex of household-head, religion, principal source of household income, household head’s highest level of education, occupation, and indicator variable for presence of any chronic diseases of any household member in last 12 months. *District*_*id*_ controls for regional fixed effects, which takes the value 1 if household *i* resides in administrative district *d*, and 0 otherwise. ε_ij_ is the idiosyncratic error term. We assume that households within a district face similar prices for respective commodities.

The system in Eq (5) was estimated using Seemingly Unrelated Regression (SUR) technique. Miscellaneous consumption category was dropped from the system of equations to meet summation restrictions. Following Banks, Blundell, & Lewbel [23] ln M and (ln M)^2^ were instrumented by natural log of monthly household income, and its squared respectively.

$$w_{ij}=\beta_{0}+\sum_{t=1}^{3}\lambda_{t}T_{it}+\beta_{2}\ln M_{i}+\beta_{3}{\left(\ln M_{i}\right)}^{2}+X\beta_{4}+\sum_{d=1}^{63}\gamma_{d}District_{id}+\varepsilon_{ij}$$ (6)

The crowding-out effects for the mutually exclusive smoking-only, smokeless-only, and both smoking and smokeless user households are estimated using Eq (6), where *T*_*it*_ denotes *t* type of tobacco consumption for household *i*, and it takes the value 1 if tobacco consumption type is *t*, and 0 otherwise. The coefficients β_1_ in Eq (5) and λ_t_ in Eq (6) estimate the percentage point differences in expenditure shares between households with and without tobacco expenditure, and therefore captures the crowding out effect.

## Results

### Unadjusted differences in expenditure share

Unadjusted differences in expenditure shares between tobacco user and non-user households, expressed as percentage points are reported in Table 4. Tobacco non-user households being the reference category, a positive percentage point difference implies that households spending on any tobacco (or on a particular type of tobacco), on average, allocated a greater share to that consumption category than non-user households; and a negative percentage point difference implies that tobacco user households allocated a smaller share.

**Table 4:** Unadjusted differences in consumption share.

	Household types by tobacco use				
	No Tobacco (n = 3,550)	Any tobacco (n = 8,690)	Smoking-only (n = 2,063)	Smokeless-only (n = 3,281)	Dual/both tobacco (n = 3,346)
	Mean share (%)	Difference (% point)	Difference (% point)	Difference (% point)	Difference (% point)
Food	55.142 (54.224,56.061)	4.475*** (3.601, 5.348)	4.254*** (3.291, 5.217)	2.825*** (1.780, 3.870)	6.239*** (5.167, 7.312)
Clothing	6.226 (5.924,6.529)	-0.564*** (-0.834, -0.294)	-0.274* (-0.551, 0.002)	-0.639*** (-0.932, -0.347)	-0.679*** (-0.983, -0.375)
Housing	10.448 (9.810,11.085)	-2.628*** (-3.208, -2.049)	-1.897*** (-2.597, -1.197)	-2.033*** (-2.716, -1.351)	-3.689*** (-4.387, -2.992)
Education	4.862 (4.411,5.312)	-1.165*** (-1.580, -0.751)	-1.228*** (-1.704, -0.752)	-0.653** (-1.099, -0.208)	-1.628*** (-2.095, -1.161)
Medical	2.989 (2.768,3.210)	0.772*** (0.533, 1.012)	0.524** (0.136, 0.912)	0.888*** (0.613, 1.162)	0.821*** (0.517, 1.125)
Life-style and hygiene	3.159 (3.078,3.240)	0.040 (-0.043, 0.124)	0.093* (-0.011, 0.198)	0.042 (-0.060, 0.144)	0.005 (-0.100, 0.109)
Energy	7.321 (7.098,7.544)	-0.326** (-0.550, -0.103)	-0.127 (-0.426, 0.172)	-0.241* (-0.505, 0.022)	-0.540*** (-0.820, -0.259)
Transport and Communication	5.559 (5.295,5.822)	-0.715*** (-0.988, -0.441)	-0.616*** (-0.955, -0.276)	-0.794*** (-1.121, -0.468)	-0.701*** (-1.046, -0.356)
Entertainment	0.588 (0.517,0.659)	0.055 (-0.022, 0.133)	0.153** (0.043, 0.262)	0.032** (-0.058, 0.123)	0.015 (-0.090, 0.120)
Miscellaneous	3.707 (3.394,4.019)	0.056 (-0.283, 0.396)	-0.882*** (-1.279, -0.484)	0.576 (0.151, 1.000)	0.157 (-0.280, 0.594)

Note

*** p<0.01

** p<0.05

* p<0.1. 95% confidence interval in the parenthesis.

Table 4 reveals statistically significant differences expenditure allocations between tobacco user and non-user households. The ‘mean shares’ in second column are the expenditure shares for the tobacco non-user households, and the last four columns show the percentage point differences from the corresponding mean shares of the tobacco non-user households. For instance, a household reporting expenditure on any tobacco, on average allocated 2.63 percentage point less on housing compared to a typical tobacco non-user household that spent 10.4% of its budget on housing. Compared to a tobacco non-user household, tobacco user (any tobacco) households, on average, allocated less on clothing, education, energy, and transport and communication. In contrast, any tobacco user households allocated more of the household total expenditure on food and medical expenses.

Albeit difference in magnitudes, a more or less similar pattern is evident for smoking-only, smokeless-only, and dual tobacco user households. In addition to food and medical expenses, the smoking-only and smokeless-only households also allocated 0.153 and 0.032 percentage points more of their household budget on entertainment. The largest differences were observed for the households reporting expenditure on both smoking and smokeless tobacco (i.e. dual tobacco).

**Fig 1:**
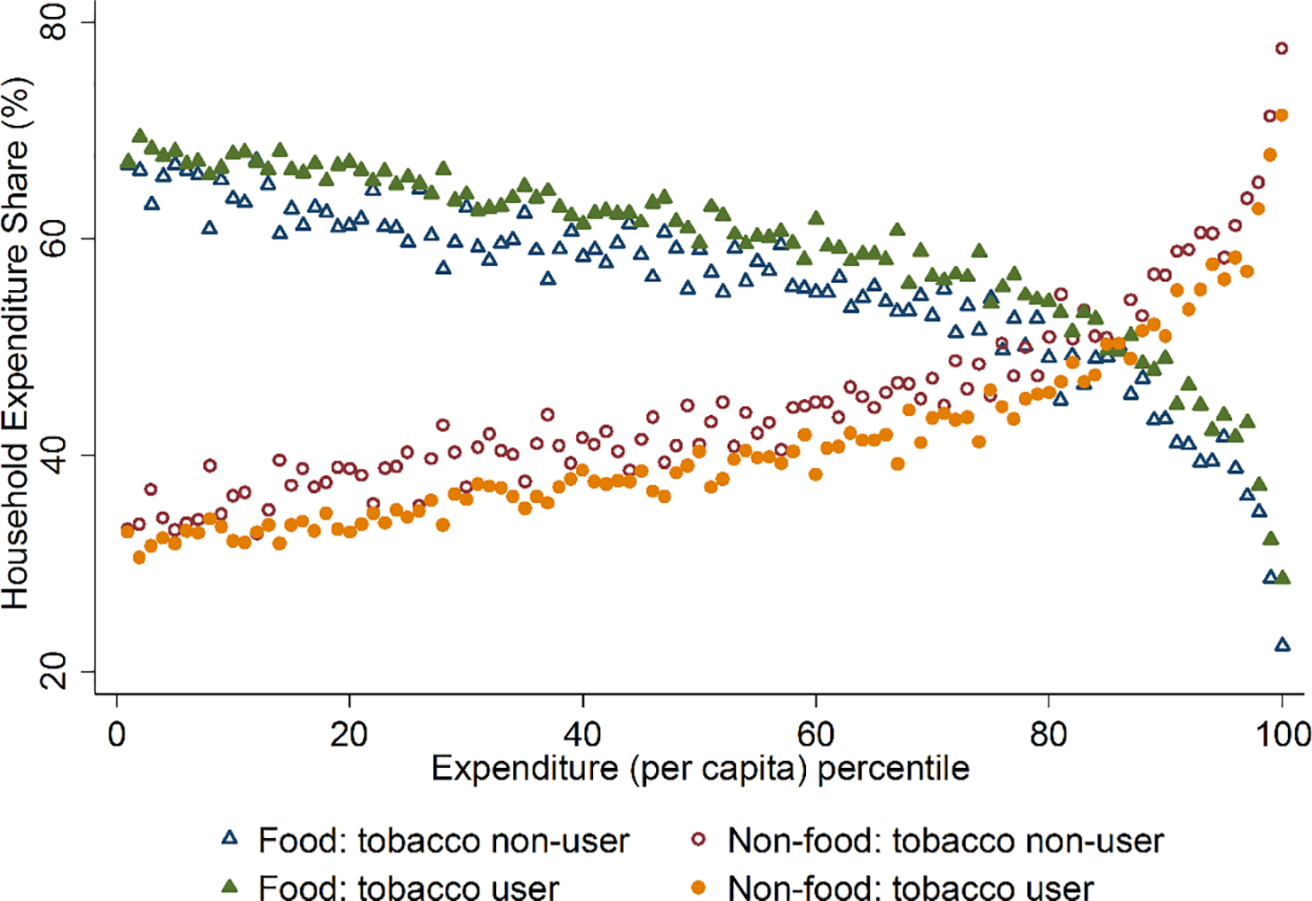
Differences in food and non-food expenditure shares between tobacco user and non-user households.

Previous studies supported crowding-out effect of tobacco expenditure on food expenditure, in contrast to what is observed in Table 4 (e.g. [14, 15]). However, for poor households, the expenditure on food and medical expenses may be less discretionary in nature, rather necessary expenditure items constituting large budget share. Therefore, the crowding-out effects are manifested in other expenditure items of discretionary nature [13]. In Bangladesh, a closer look at the food and non-food expenditure pattern by household expenditure per capita percentiles in Fig 1 revealed that expenditure share allocated to food were higher for households that reported any tobacco expenditure than tobacco non-user households. This was observed throughout the expenditure percentile distribution. Accordingly, tobacco user households allocated less budget on non-food items than the tobacco non-user counterparts, throughout the percentile distribution.

### Adjusted differences in expenditure share

The unadjusted differences in expenditure shares presented in the previous section did not control for other household socio-economic characteristics that may affect budget allocation. We implemented Eqs (5) and (6) to control for these characteristics. Table 5 reports the coefficients of the dichotomous tobacco consumption variable for each budget share equation in the system. The coefficient values are the average percentage point differences in the budget shares for the corresponding expenditure categories; a negative coefficient indicates a lower budget allocation among tobacco-user households than non-user households.

**Table 5:** Adjusted differences in expenditure share.

	Household types by tobacco use				
	No Tobacco (n = 3,545)	Any tobacco (n = 8,644)	Smoking-only (n = 2,050)	Smokeless-only (n = 3,264)	Dual/both tobacco (n = 3,330)
	Mean share (%)	Difference (% point)	Difference (% point)	Difference (% point)	Difference (% point)
Food	54.42 (53.94,54.90)	2.383*** (1.916,2.850)	2.118*** (1.520,2.716)	1.993*** (1.442,2.545)	3.264*** (2.669,3.859)
Clothing	6.44 (6.33,6.56)	-0.410*** (-0.524,-0.296)	-0.332*** (-0.478,-0.186)	-0.404*** (-0.538,-0.269)	-0.510*** (-0.655,-0.365)
Housing	10.21 (9.88,10.54)	-0.788*** (-1.144,-0.431)	-0.420* (-0.877,0.037)	-0.779*** (-1.200,-0.358)	-1.230*** (-1.684,-0.775)
Education	4.94 (4.69,5.19)	-0.543*** (-0.795,-0.291)	-0.680*** (-1.003,-0.357)	-0.167 (-0.465,0.131)	-0.934*** (-1.256,-0.613)
Medical	3.19 (3.02,3.37)	0.187* (-0.029,0.402)	0.168 (-0.110,0.445)	0.176 (-0.08,0.431)	0.225 (-0.051,0.500)
Life-style and hygiene	3.24 (3.19,3.29)	-0.0319 (-0.095,0.031)	0.024 (-0.057,0.105)	-0.082** (-0.157,-0.007)	-0.023 (-0.104,0.057)
Energy	7.42 (7.29,7.54)	-0.170*** (-0.297,-0.042)	-0.139* (-0.302,0.025)	-0.212*** (-0.363,-0.062)	-0.143* (-0.306,0.019)
Transport and Communication	5.54 (5.36,5.71)	-0.402*** (-0.619,-0.184)	-0.330** (-0.610,-0.051)	-0.470*** (-0.728,-0.213)	-0.385*** (-0.663,-0.107)
Entertainment	0.64 (0.58,0.70)	0.0223 (-0.056,0.100)	0.063 (-0.037,0.163)	0.01 (-0.083,0.102)	-0.007 (-0.106,0.093)

*** p<0.01

** p<0.05

* p<0.1. 95% confidence interval in the parenthesis.

The adjusted differences in expenditure shares for ‘any’ tobacco user households confirm that household tobacco use crowds-out budget allocations in clothing, housing, education, energy, and transport and communication. The budget allocations in a tobacco user household are higher for food and medical expenses, although the coefficients of medical expenses when generated separately for smoking-only, smokeless-only, and dual tobacco use households were not statistically significant. The adjusted differences for food, clothing, housing, and education are relatively larger (in absolute terms) for dual tobacco user households, than the adjusted differences of those for any-, smoking only-, or smokeless only- tobacco user households.

Spending patterns and tobacco consumption could vary across rural and urban areas, and across different level of consumption expenditure. Table 6 presents the adjusted differences in expenditure shares by rural and urban households and for bottom and top expenditure quintiles. We observe crowding-out effects of tobacco consumption at different sub groups as well, however, the magnitudes become different. For example, all smokeless-only households on average allocated 0.33 percentage points less in clothing than all non-tobacco households, while smokeless-only households in rural bottom quintile allocated 1.04 percentage points less than non-tobacco households in rural bottom quintile. These results provide a more comprehensive perspective of the crowding-out effect.

**Table 6:** Adjusted differences (percentage point) in expenditure share by rural and urban and by bottom and top expenditure quintiles.

	Food	Clothing	Housing	Education	Medical	Hygiene	Energy	Transport and Communication	Entertainment
**Any Tobacco**									
Rural bottom	1.743***	-0.935***	-0.731***	0.252	0.673***	0.0761	-0.115	-0.555**	-0.101**
Rural top	2.120**	-0.22	-1.898**	-0.335	0.147	-0.124	-0.0226	-1.328***	0.316*
Urban bottom	2.327***	-0.502**	-0.712*	-0.557**	0.134	-0.0193	0.380*	-0.862***	-0.128
Urban top	3.435***	-0.0534	-1.951	-1.346	0.088	-0.13	-0.0694	0.587	-0.134
**Smoking only**									
Rural bottom	1.876**	-0.602**	-0.45	-0.218	0.404	-0.0287	0.0858	-0.617**	-0.0604
Rural top	3.082**	0.241	-1.291	-0.542	0.602	0.0567	0.322	-1.265*	0.648**
Urban bottom	1.929**	-0.539*	0.26	-0.341	0.054	0.0804	0.00486	-1.045***	-0.143
Urban top	2.831**	-0.176	-2.803*	-2.328*	1.22	-0.0596	-0.106	0.321	0.141
**Smokeless only**									
Rural Bottom	1.965**	-1.044***	-0.831***	0.262	0.771***	0.126	-0.304	-0.689**	-0.124**
Rural Top	1.286	-0.308*	-1.508	0.196	0.0917	-0.205	-0.111	-1.827***	0.211
Urban Bottom	1.905**	-0.24	-1.330***	-0.486	0.129	-0.121	0.924***	-0.717**	-0.223*
Urban Top	3.403***	0.0357	-0.207	-0.396	-0.861	-0.136	-0.0595	0.448	-0.298
**Dual/Both tobacco**									
Rural Bottom	1.405*	-1.092***	-0.854***	0.623**	0.789***	0.109	-0.08	-0.366	-0.111**
Rural Top	2.983***	-0.370*	-3.009***	-1.154**	-0.0587	-0.0981	-0.0936	-0.468	0.282
Urban Bottom	3.152***	-0.708**	-1.180**	-0.856***	0.224	-0.0312	0.273	-0.802***	-0.024
Urban Top	4.570***	-0.0448	-4.501**	-1.827	0.304	-0.241	-0.0275	1.378	-0.235

*** p<0.01

** p<0.05

* p<0.1. The confidence intervals are not reported in the table.

## Discussion

The deleterious effects of tobacco consumption, including smoking tobacco, smokeless tobacco, and passive smoking, on health is well-documented [1, 2, 3]. The tobacco-attributable deaths, disabilities, and diseases cause enormous economic tolls on individuals, households, and society. These economic costs are described as direct medical costs for the individuals and families, and consequent strain on the national level healthcare finances, as well as indirect costs due to absenteeism and presenteeism for the earners and informal caregivers, constrained education attainments school attendance and learning, and consequent lost productivity at the macro level. Another mechanism through which tobacco use can negatively impact well-being of tobacco users and family members is due to displacement of consumption of other basic necessities due to expenditure on tobacco, generally described in the literature as the ‘crowding-out effect’ [13]. Given fixed household budget, the displacements often occur for items that constitute human capital investments (e.g. food and nutrition, education, health and hygiene, housing etc.) rendering life-course implications on household well-being.

The existing tobacco expenditure crowding-out literature mainly highlighted the consumption displacement effects for smoking tobacco only. Apparently, the expenditure on smokeless tobacco in those literature was included into one of the consumption categories other than tobacco, most probably as part of the food consumption item. This paper examined how the household expenditure patterns for smoking-, smokeless-, and dual- (both smoking and smokeless) user households differ from tobacco non-user households, using household level data from Bangladesh, and provided additional policy insights for much needed different tobacco-use types. The analyses on smokeless and dual tobacco use constitute a major contribution of this paper in the tobacco crowding-out literature.

The econometric analysis showed that tobacco user households on average allocate less in clothing, housing, education, energy, and transportation and communication compared to tobacco non-user households. Mean expenditure share of food and medical expenditure of tobacco user households, however, are greater than those of tobacco non-user households. Albeit similar patterns were observed for different types of tobacco user households, there are differences in magnitudes depending on the type of tobacco-use, rural-urban locations and economic status.

The greater share of medical expenditure of tobacco user households could be linked to tobacco-attributable diseases and related health costs. Tobacco use is a major risk factor for various noncommunicable diseases and the direct cost (healthcare expenditures) of smoking in low-income and lower-middle-income countries was estimated around 4% of the total health expenditure in 2012 [24]. Hence, tobacco user households might suffer from greater disease burden, resulting in larger budget share of medical expenses.

Findings from the crowding-out literature demonstrate the importance of the economic effects of higher spending on tobacco on household standards of living and expenditure patterns and these effects may be different in different countries [8,10,11,20]. On the association between smoking tobacco and food expenditure, the crowding-out effect literature suggests mixed results. For instance, Chewla and Walbeek [15], Wang et al. [11], San and Chaloupka [14], and John [10] found crowding-out of food expenditure for Zambia, China, Turkey, and India, respectively. On the other hand, the studies by Do and Bautista [13] on low and middle income countries and John et al. [16] on Cambodia did not find crowding-out effect for food consumption. Other literature suggest that tobacco user households may crowd-out expenditure on quality or nutrition-rich food [25, 26, 27, 28, 29, 30]. In our study, we found that food expenditure shares, in general, were higher for tobacco user households than tobacco non-user households throughout the consumption expenditure distribution. In Bangladesh, after deducting the tobacco expenditure from the total expenditure, a household on average spends between 55% to 61% of their budget on food, depending on household tobacco use status. Therefore, the expenditure on food may be less discretionary in nature. Further research on the macro-nutrient level food consumption pattern may reveal important differences between tobacco user and non-user households.

Our findings reaffirm the negative impact of tobacco expenditure on human capital investments, impeding short- and long-term economic well-being potentials at the household level, and in effect, for the society. Policy measures that reduce tobacco use could reduce displacement of commodities by households with tobacco users, including those commodities that can contribute to human capital investments.
